# Giant Persistent Photoconductivity of the WO_3_ Nanowires in Vacuum Condition

**DOI:** 10.1007/s11671-010-9800-1

**Published:** 2010-09-30

**Authors:** Kai Huang, Qing Zhang

**Affiliations:** 1 School of Electrical and Electronic Engineering, Microelectronics Center, Nanyang Technological University, Singapore, 639798, Singapore

**Keywords:** WO_3_ nanowire, Persistent photoconductivity

## Abstract

A giant persistent photoconductivity (PPC) phenomenon has been observed in vacuum condition based on a single WO_3_ nanowire and presents some interesting results in the experiments. With the decay time lasting for 1 × 10^4^ s, no obvious current change can be found in vacuum, and a decreasing current can be only observed in air condition. When the WO_3_ nanowires were coated with 200 nm SiO_2_ layer, the photoresponse almost disappeared. And the high bias and high electric field effect could not reduce the current in vacuum condition. These results show that the photoconductivity of WO_3_ nanowires is mainly related to the oxygen adsorption and desorption, and the semiconductor photoconductivity properties are very weak. The giant PPC effect in vacuum condition was caused by the absence of oxygen molecular. And the thermal effect combining with oxygen re-adsorption can reduce the intensity of PPC.

## Introduction

One-dimensional (1D) nanotubes, nanowires, or nanorods have shown much higher sensitivity than bulk materials at room temperature because of their higher surface-to-volume ratio and stronger dependence of electrical conductance on the amount of adsorbates [1-5]. Their optical and electrical characterization is a direct way to gain a deep comprehension of some of novel phenomena of the nanostructure that originate from the overexposure of the bulk of nanomaterials to surface effects. Recently, the persistent photoconductivity (PPC) effect has been observed in ZnO nanowire [6], n-type GaN thin film [7], and rough Si nanomembranes [8]. Persistent photoconductivity, which means that photoconductivity persists after the illumination has ceased and hindered the quick recovery of the initial unperturbed state, implies interesting applications in bistable optical switches [9,10] and radiation detectors [11,12].

Many methods are used to investigate the origin of PPC, including photoluminescence [13], optical absorption [14], photoconductivity [15], and PPC measurements [16]. The kinetic mechanisms of PPC experiments are proposed by several groups. Some claims that this PPC phenomenon is related to metastable bulk defects located between shallow and deep energy levels. According to this assumption, oxygen vacancies can be excited to a metastable charged state after a structural relaxation [17]. And others demonstrate that the PPC state is directly related to the electron–hole separation near the surface. The surface built-in potential separates the photo-generated electron–hole pairs and accumulates holes at the surface. After illumination, the charge separation makes the electron–hole recombination difficult and originates PPC [7]. And the thermal and electric field effects have also been reported to reduce the intensity of the PPC [6,7], simultaneously. However, there is no a widely accepted mechanism has been presented.

In this paper, we fabricated a single WO_3_ nanowire device and presented a systematic study on giant PPC effect in vacuum condition. In addition, WO_3_ nanowire as a UV photodetector has been reported by our previous results [18]. And no any decay current can be observed in absence of oxygen molecular atmosphere, and a gradually decay current can only be presented in air condition. The WO_3_ nanowire coated with 200 nm SiO_2_ layer can obviously reduce the photoresponse of the device. Moreover, the thermal and electric field effects cannot accelerate the decay current in vacuum condition. Based on these results, we thus conclude that the photoconductivity of WO_3_ nanowire is only related to the oxygen adsorption and desorption, the semiconductor photoconductivity of WO_3_ nanowire is very weak when compared to the surface effect, and the intensity of PPC effect is directly related to the oxygen molecular re-adsorbed rate.

## Experimental Section

**Figure 1:**
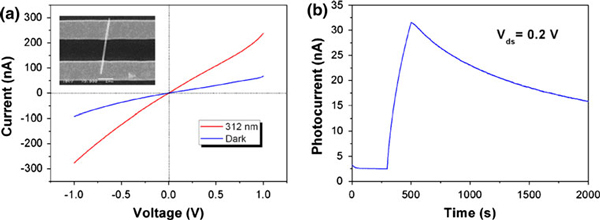
**a The *I*_ds_–*V*_ds_ characteristics of a single WO_3_ nanowire under dark and 312-nm UV illumination (~1 mW/cm^2^)**. The inset is an SEM image of a single WO_3_ nanowire device. **b** The persistent photoconductivity of the WO_3_ nanowire with *V*_ds_ = 0.2 V.

The WO_3_ nanowires were synthesized using a simple hydrothermal method in our previous reports [19]. Tungsten powder and hydrogen peroxide were used as reactive materials, and the Na_2_SO_4_ was added to the solution as catalyst. Then the solution was sealed in autoclave and maintained at 180°C for 12 h. At last, high-purity WO_3_ nanowires were obtained. To characterize the photoelectrical properties of the WO_3_ nanowire, a single nanowire was assembled into field-effect transistor (FET) device using a standard photolithography. A parallel Ti/Au (10/200 nm) electrodes spaced about 2 μm apart were fabricated on a single WO_3_ nanowire, as shown in inset of Figure 1a. The UV photoconductivity measurements were performed under atmospheric and room temperature conditions with UV illumination (Spectroline handheld E-Series) and Agilent B1500A semiconductor Device Analyzer. The *I*_ds_–*V*_ds_ curves of the nanodevices under dark and 312-nm UV illumination (~1 mV/cm^2^) were shown in Figure 1a. Under the dark condition, the nonlinear *I*–*V* characteristics reflect a back-to-back diode device. The current can increase from ~100 to ~300 nA after 200-s UV illumination.

In Figure 1b, the photocurrent can increase to ~30 nA with *V*_ds_ = 0.2 V. However, no saturated photocurrent can be obtained, which maybe caused by the incomplete desorption of oxygen species on the surface of WO_3_ nanowire, similar to the ZnO nanowire as UV photodetector in Zhou's reports [20]. The current is still about 17 nA after switching off the UV light more than 1.5 × 10^3^ s, cannot recover to initial 2.5 nA, as shown in Figure 1b. That demonstrates the existence of obviously persistent photoconductivity in WO_3_ nanowire. With the decay time lasting to 2 h or longer, the current cannot back to the initial states.

## Results and Discussion

**Figure 2:**
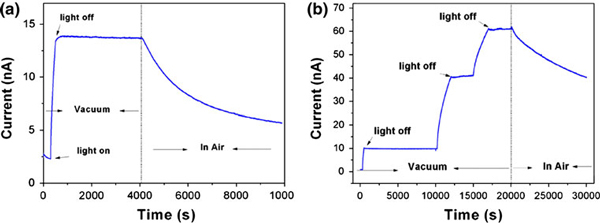
**a The persistent photoconductivity of the WO_3_ nanowire device under vacuum and in air conditions**. **b** The persistent photoconductivity under discontinuous UV illumination. All the biases are 0.1 V.

In order to observe the persistent photoconductivity of the WO_3_ nanowire in vacuum condition, we designed a vacuum chamber with a quartz glass window, which allows the UV illumination reach to the devices. When switching off the UV light in vacuum (0.1 mbar), the current can preserve a constant state (~13.5 nA) and hold more than 3.5 × 10^3^ s without any decay, which presents a giant persistent photoconductivity phenomenon, as shown in Figure 2a. When the decay time was extended to 10^4^ s, no decay current could be observed as shown in the first light off Figure 2b. However, once opening the chamber to air condition, a gradual decreasing current can be only presented, as shown in right side of the Figure 2a, b. It is noted that the duration of UV illumination is more than 3 × 10^3^ s, and no saturated photocurrent can be observed as shown in Figure 2b.

**Figure 3:**
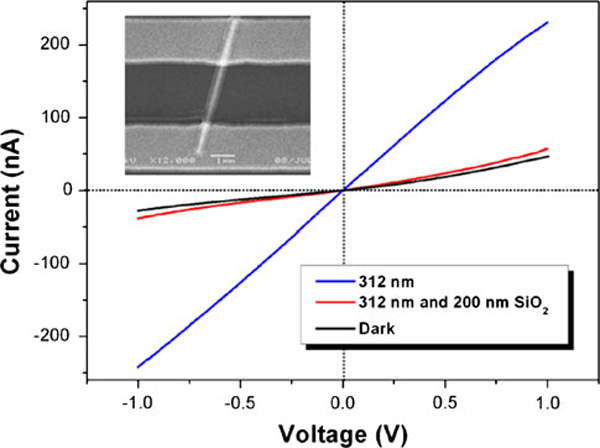
**The *I*_ds_–*V*_ds_ curves of the device coated with SiO_2_ under dark (*black curve*) and 312-nm illumination (*red curve*) and without SiO_2_ coating under 312-nm UV illumination (*blue curve*), respectively**. Inset is the device coated with 200-nm SiO_2_ layer.

To analyze the semiconductor properties of WO_3_ nanowire for the photoconductivity, a 200-nm SiO_2_ layer was deposited on devices using PECVD at 200°C to isolate the effects of oxygen absorption and surface defects. In addition, SiO_2_ was also demonstrated to be effective in surface passivation of nanostructures [21]. A transparent SiO_2_ layer coating with the WO_3_ nanowire can be seen from the inset SEM image of Figure 3. No photocurrent can be observed in a control device, which is only coated with the same SiO_2_ layer between the two electrodes without any nanowire. With 200-nm SiO_2_ layer coating, the photoresponse almost disappeared as shown in Figure 3 (red curve), which is smaller than that of before coating (blue curve).

Based on the semiconductor theory, UV photons can generate electron–hole pairs in the bulk of the nanowires. The photoresponse (Δ*G*_ph_) reaches a steady state in which the recombination and the generation rates are equal. Here, the photoresponse was defined as:

(1) $$\Delta G_{\text{ph}}=G_{1}-G_{0}$$

where *G*_0_ was the initial value in darkness, and *G*_1_ was the value after switching off light. However, some authors claim the existence of two different mechanisms that steer the photoresponse for metal oxides. The former one is a fast band-to-band recombination (semiconductor characteristics) in their bulk with characteristic times in the nanosecond range [22]. The latter becomes dominant in nanostructure materials, which is highly dependent on the existence of chemisorbed oxygen molecules at their surfaces, and holes can discharge oxygen species from the surface by indirect electron–hole recombination mechanism. Thus, the change numbers of n and p carriers (Δn and Δp) can be given by [6,23]

(2) $$\Delta G_{\text{ph}}\propto \Delta n=\Delta p=\frac{g}{1\text{/}t_{\text{bulk}}+1\text{/}t_{\text{surf}}}$$

where *g* is the photogeneration rate of carriers per volume unit, and *t*_bulk_ and *t*_surf_ are the lifetimes of the photocarriers recombined in the bulk and at the surface. In Figure 3, the SiO_2_ layer can suppress the oxygen adsorption at the surface of WO_3_ nanowire, and the photoresponse is only decided by the *t*_bulk_. But no obvious photoresponse can be observed. It implies that the recombination of photo-generated electron and hole pairs is completely dominated by the oxygen adsorption mechanism in the WO_3_ nanowires, and the band-to-band recombination mechanism from the WO_3_ nanowire can be neglected. In air environment, a ΔG_ph_ values (72 nS in Figure 1b) is smaller than that of in vacuum condition (112 nS in Figure 2a, 600 nS in Figure 2b).

As an indirect gap semiconductor, WO_3_, the recombination of electrons and holes is through a recombination center (*E*_*t*_) between the valence band and conduction band. The adsorbed oxygen molecular can be served as the recombination center at the surface of nanowire. Because of the absence of oxygen molecular in vacuum condition, the recombination of electrons and holes assisted by surface recombination center (adsorbed oxygen) cannot be occurred, and no decay current can be observed. So, only holes accumulate near the surface can recombine with electrons at the oxygen-assisted mechanism, which can explain the giant PPC phenomenon of WO_3_ nanowire in vacuum condition. Once the air is pumped into the vacuum chamber, oxygen species gradually re-adsorbed on the surface and captured these electrons, which results in a slow current decay in air condition.

**Figure 4:**
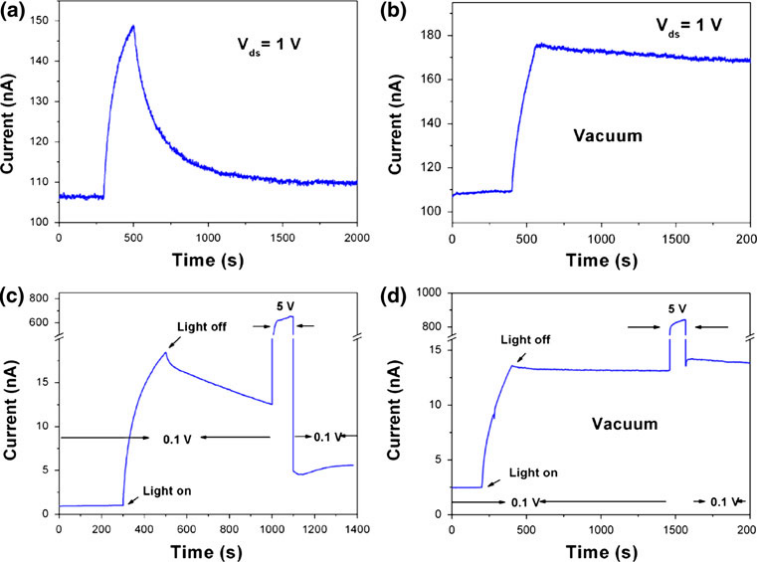
**The photoresponse with bias 1 V in **a** air and **b** vacuum condition**. The persistent photoconductivity with 5-V pulse in **c** air and **b** vacuum condition. The bias is 0.1 V.

How to reduce the intensity of PPC? Recently, a high bias and a pulse electric field effects have been reported to accelerate the decay process [6,7]. For the high bias effect, carriers gain thermal energy from high bias can easily overcome the built-in potential and accelerate the recombination photo-generated electron and hole pairs. For the pulse electric field effect, it will enlarge the capture cross-section of hole traps and increase the recombination rate. The similar results have also been presented for the WO_3_ nanowires. When we used a *V*_ds_ = 1 V and switched off UV light, a faster decay current can be found as shown in Figure 4a. At the same time, a 5-V pulse with 100 s can lead to a sudden decreasing current as shown in Figure 4c.

It is very interesting that we observed different phenomenon between in air and vacuum conditions. With the *V*_ds_ = 1 V and switching off the UV light in vacuum, the current is in a constant state similar to that of the low bias *V*_ds_ = 0.1 V shown in Figure 2a. Increasing the bias cannot accelerate the decay process in vacuum condition. Similarly, a five pulse voltage could not change the current as shown in Figure 4d. Here, whatever high bias or high electric field is applied, no decay current can be observed in vacuum condition. So, the thermal effect and electric field mechanisms fail to explain the phenomenon.

Based on the results, we can conclude that under no high bias or high bias condition, the oxygen molecular always acts as a key role to decrease the current. In air condition, the higher current caused by high bias can increase the concentration of carriers and enlarge the conduction channel along the nanowires, and the more electrons can easily cross the depletion layer near the surface of nanowire and combine with oxygen molecular, which reduces the electrical conductance of WO_3_ nanowire. So, a "sudden" dropping current can be found when switching to a low bias as shown in Figure 4c. Opposite, there is an absence of oxygen molecular in vacuum condition as the recombination centers to decrease the current as shown in Figure 4d. Thus, a mechanism, combination of high bias and oxygen adsorption at the surface of WO_3_ nanowire, can perfectly explain the phenomenon.

## Conclusions

In summary, we have observed a giant PPC phenomenon of WO_3_ nanowire in vacuum condition. No decreasing current can be observed in absence of oxygen molecular atmosphere, and a gradually decay current can be presented in air condition. For the SiO_2_-surrounded WO_3_ nanowire, there is a very weak photoresponse in our measurements. The high bias and high electric field effects can accelerate the decay process in air, but not in vacuum condition. We can conclude that: (1) the photoconductivity of WO_3_ nanowire is mainly related to the oxygen adsorption and desorption, and the typical semiconductor photoconductivity properties of WO_3_ nanowire are very weak comparing to the surface effect; (2) the giant PPC effect is caused by the absence oxygen molecular as recombination center in vacuum condition, and the intensity of PPC is only depended on the oxygen molecular re-adsorbed rate on the surface of WO_3_ nanowires; (3) the thermal effect and oxygen re-adsorption can accelerate the decay current.
